# Preliminary evaluation of a novel method for computed tomography quantification of lumbosacral articular process displacement in dogs with and without degenerative lumbosacral stenosis

**DOI:** 10.3389/fvets.2024.1436299

**Published:** 2024-08-05

**Authors:** Oscar Carballo, Steven W. Frederick, Deborah A. Keys, Sarah A. Moore, James T. Giles

**Affiliations:** ^1^BluePearl Pet Hospital, San Antonio, TX, United States; ^2^BluePearl Science, Tampa, FL, United States; ^3^Kaleidoscope Statistics, LLC, Athens, Georgia

**Keywords:** degenerative lumbosacral stenosis, computed tomography, lumbosacral, lumbosacral articular process displacement, dynamic CT

## Abstract

**Objective:**

This study aimed to describe the diagnostic discrimination and reliability of a novel technique for quantifying lumbosacral articular process displacement (LSAPD) on dorsal plane computed tomography (DPCT) imaging in dogs with and without degenerative lumbosacral stenosis (DLSS).

**Study design:**

DPCT surveys of the lumbosacral vertebral column were performed with dogs positioned in extension and flexion. LSAPD is defined as the distance between the cranial aspects of the L7 and S1 articular processes. The LSAPD ratio is identified by dividing the LSAPD by the length of the L7 articular process. Intraclass correlation coefficients (ICCs) for intra- and inter-observer reliability were calculated, and logistic regressions were used to test for the association of LSAPD and LSAPD ratio with odds of DLSS. Significance was set at 0.05. Receiver operator characteristic (ROC) curves were calculated to determine diagnostic discrimination and optimal cutoff for LSAPD and LSAPD ratio in the diagnosis of DLSS.

**Results:**

Intra- and inter-observer reliabilities were excellent for most measurements. In the current cohort, excluding covariates, the area under the curve (AUC) (95%CI) for LSAPD and LSAPD ratio measured in a flexed position were both 0.89 (0.82–0.96), suggesting potentially excellent discrimination for using this measurement as a marker for diagnosing DLSS, pending further studies. The cutoffs for flexed LSAPD and LSAPD ratio that maximizes Youden’s index were ≥ 1.2 mm and ≥ 9%, respectively. When age and weight were subsequently included as covariates in a multivariable analysis, a significant relationship between LSAPD or LSAPD ratio and odds of diagnosis of DLSS was not demonstrated, suggesting the need for a larger sample size.

**Conclusion:**

The results of this study suggest that measurements of LSAPD and LSAPD ratio on DPCT are feasible and reliable, although their diagnostic discrimination in DLSS should be evaluated further in future prospective studies.

## Introduction

Degenerative lumbosacral stenosis (DLSS) is a multifactorial condition affecting the lumbar vertebral column primarily in medium- to large-breed and military working dogs, with German Shepherd dogs (GSDs) being predisposed (1–5). Lumbosacral instability appears to play a significant role in some dogs and is a major risk factor for DLSS in military working dogs due to their extensive mobility of the LS region in flexion, extension, and axial rotation (1, 2, 6, 7). Dogs with instability may be more susceptible to injuries to the LS soft tissues, including the joint capsule of the articular process, ligamentum flavum, dorsal longitudinal ligaments, and intervertebral disks. These could predispose working dogs to early onset of DLSS and removal from active-duty status (1–5). The spinal articular processes are highly innervated, and stretching of their joint capsule, as caused by displacement, has been demonstrated as a major source of lower back pain in people (8–12), which could be similar to dogs. It is also a potential predisposing factor to disk degeneration in the canine lower lumbar spine, which causes further instability (13).

Computed tomography has been used to characterize DLSS and LS instability in dogs (1, 2, 4, 6, 7, 13–23). Henninger et al. reported a two-part study on CT that offers a very detailed anatomical description of the lumbosacral region in flexion and extension using a soft tissue and bone window, which could help provide qualitative information on LS instability causing DLSS (14, 15), except it is lacking quantitative information. Seiler et al. and Benninger et al. evaluated lumbar and lumbosacral articular process angles and tropism, reporting that articular process joint angles were higher at L7-S1 in GSDs, which could predispose the breed to disk degeneration due to a higher axial rotation instability (7, 13). However, Benninger et al. identified that articular process joint tropism was higher in GSD compared to the control group, and no correlation with disk degeneration was identified. They found that the total range of motion for flexion and extension was mainly associated with the spinal articular process angle, disk height, and lever arm length (7). Although the study provided useful biomechanical information, it was performed on cadaveric spines, and therefore its association with clinical signs is unknown. Worth et al., Jones et al., and Higgins et al. reported on the quantitative measurement of the intervertebral neurovascular foraminal volume (16–18) and concerns encountered about the repeatability of the measurements across all three studies, specifically inter-observer repeatability. Saunders et al. evaluated the rostral projection of the sacral lamina as a component of DLSS in GSDs, 125 police dogs, and 18 pet dogs and found no association with the diagnosis of DLSS (4). Jones et al. performed a quantitative CT study on the size of the paraspinal muscles in flexion and extension about LS instability (change in the foraminal area) and found no correlation between the two (19). The previously mentioned studies provide important information for the evaluation of the LS spine in dogs; however, there is still quantitative ambiguity as to what defines LS instability in correlation with imaging findings.

If LSAPD is associated with LS instability, then quantification of LSAPD could be useful as a diagnostic tool to help correlate clinical signs of lumbosacral instability with structural changes, predict longevity in military and other working dogs, make recommendations for adjustment to activity level in active dogs with a DLSS diagnosis, and inform recommendations for medical or surgical intervention, particularly spinal stabilization. The primary objective of this study was to describe a novel method of quantifying LSAPD on dynamic DPCT imaging that could potentially be used in future studies as an imaging marker of lumbosacral articular process instability. The first working hypothesis was that LSAPD could be successfully measured on DPCT in both flexed and extended dynamic images with strong intra- and interobserver reliability. The second working hypothesis was that increased LSAPD measured in flexed images would be associated with the presence of DLSS. The third working hypothesis was that a ratio of LSAPD to L7 articular process length, used to control for differences in breed conformation, would show promise as a diagnostic imaging marker for DLSS.

## Methods

### Experimental design

This exploratory study used both a retrospective and prospective analytical design to evaluate diagnostic discrimination and repeatability of LSAPD measurements, as obtained via DPCT, in dogs with and without a clinical diagnosis of DLSS.

### Case selection

Prior to initiation, this study was reviewed and approved by the BluePearl Science Veterinary Clinical Studies Committee and Holland Military Working Dog Veterinary Hospital Institutional Animal Care Use Committee.

### DLSS group

Dogs were identified for inclusion in the DLSS group through a retrospective medical records search from January 2020 to November 2021 for dogs presenting to BluePearl Pet Hospital (Stone Oak, San Antonio, TX) and prospectively from the Holland Military Working Dog Veterinary Hospital (Lackland, TX) between September 2021 and July 2022. Medical records were reviewed by a single investigator (OC; surgery resident) and were included if they had been attributed a clinical diagnosis of DLSS by an ACVS board-certified small animal surgeon in their medical record. This diagnosis was based on a complement of lumbosacral spinal hyperpathia noted on clinical examination with or without paresis or other signs of an L7-S1 myeloradiculopathy. Dogs were also required to have had dynamic (flexion and extension) CT imaging of the lumbosacral vertebral column available for review and no other spinal disease or severe LS articular process osteophytosis identified on CT that would prevent measurement of LSAPD. The presence or absence of dynamic displacement of the lumbosacral articular process was not considered an inclusion criterion for dogs in the DLSS group.

### Control group

Healthy, neurologically, and orthopedically normal dogs were prospectively enrolled in the control group from a cohort of large-breed working dogs presented to Holland Military Working Dog Veterinary Hospital for routine health evaluations between September 2021 and July 2022. The physical exam in the control group was performed by a surgery resident (OC) with a standardized exam checklist of clinical signs that could be associated with DLSS (LS pain, pain on hyperextension of tail, iliopsoas pain, conscious proprioception deficits, and pain on extension of hip), and no behavioral assessments were performed. Control dogs underwent dynamic CT imaging of the lumbosacral vertebral column using the same standard positioning and imaging protocols used for DLSS dogs at the authors’ institution. The presence or absence of dynamic displacement of the lumbosacral articular process was not considered an inclusion criterion for dogs in the control group.

### Imaging procedure

DLSS dogs were sedated with butorphanol (0.2 mg/kg [0.09 mg/lb], IM) or, if an additional elective surgical procedure (e.g., castration and gastropexy) was scheduled, hydromorphone (0.1 mg/kg [0.05 mg/lb], IM) prior to anesthetic induction with propofol (4 mg/kg [1.8 mg/lb], IV) and maintenance with isoflurane. Control dogs were sedated with dexmedetomidine (10 mcg/kg [4.5 mcg/lb], IM) and butorphanol (0.15 mg/kg [0.07 mg/lb], IM) for imaging acquisition.

**Figure 1:**
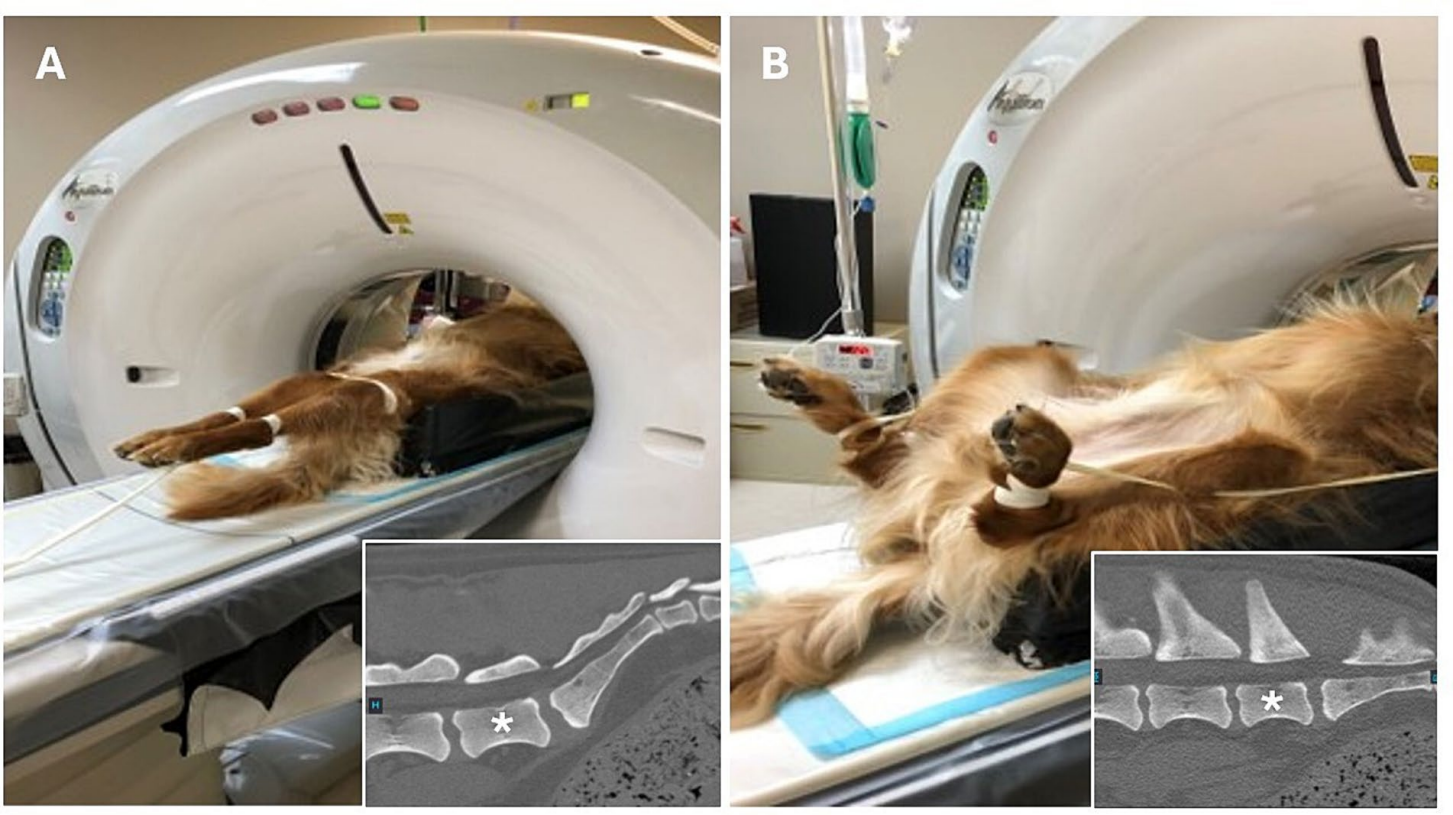
Positioning for computed tomography quantification of lumbosacral articular process displacement in dogs. Dogs were first positioned in dorsal recumbency with the pelvic limbs directed caudally and the patellas centered and pointing toward the ceiling to facilitate obtaining measurements with the lumbosacral articulation in an extended position. **(A)** After initial image acquisition, dogs were repositioned such that the pelvic limbs were fully flexed at the coxofemoral joint and stifles simultaneously to facilitate obtaining measurements with the lumbosacral articulation in a flexed position. **(B)** Insets show the orientation of the lumbosacral junction in each position, *denotes L7 vertebral body.

Once chemically restrained, all dogs (prospective and retrospective groups) were identically positioned for two computed tomography studies (Figure 1), as described by Jones et al. (17). First, dogs were positioned in dorsal recumbency with the pelvic limbs extended caudally and the patellas centered and pointing toward the ceiling. Second, dogs were positioned with the pelvic limbs flexed at the coxofemoral joint and stifle simultaneously. All imaging studies included the entire lumbosacral junction with a slice thickness of 0.5 mm for reformatting and exposure settings using a kilovoltage peak (kVp) of 120 and a milliamperage (mA) of 300. Imaging studies for DLSS dogs were performed with the Aquilion 64, 64-slice CT system (Canon Medical Systems USA, Inc., Tustin, CA), and imaging studies for control dogs were performed with the Lightspeed VCT 64, 64-slice CT system (GE Healthcare, Chicago, IL).

### Measurement technique

**Figure 2:**
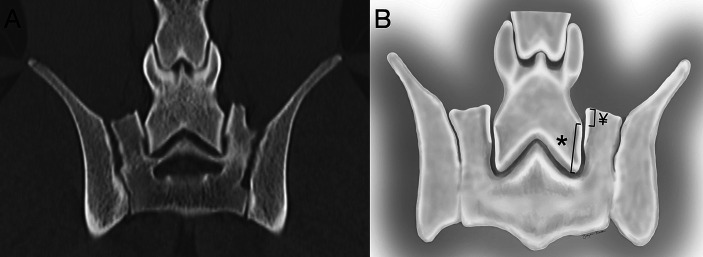
**(A)** Computed tomography image of the L7-S1 joint in the dorsal plane. **(B)** The L7 articular process was measured (mm) from the articular process’s most cranial to most distal point (*), and the lumbosacral articular process displacement was measured (mm) from the cranial point of the sacral articular process to the level of the cranial point of the L7 articular process on the sacral side of the joint (¥).

**Figure 3:**
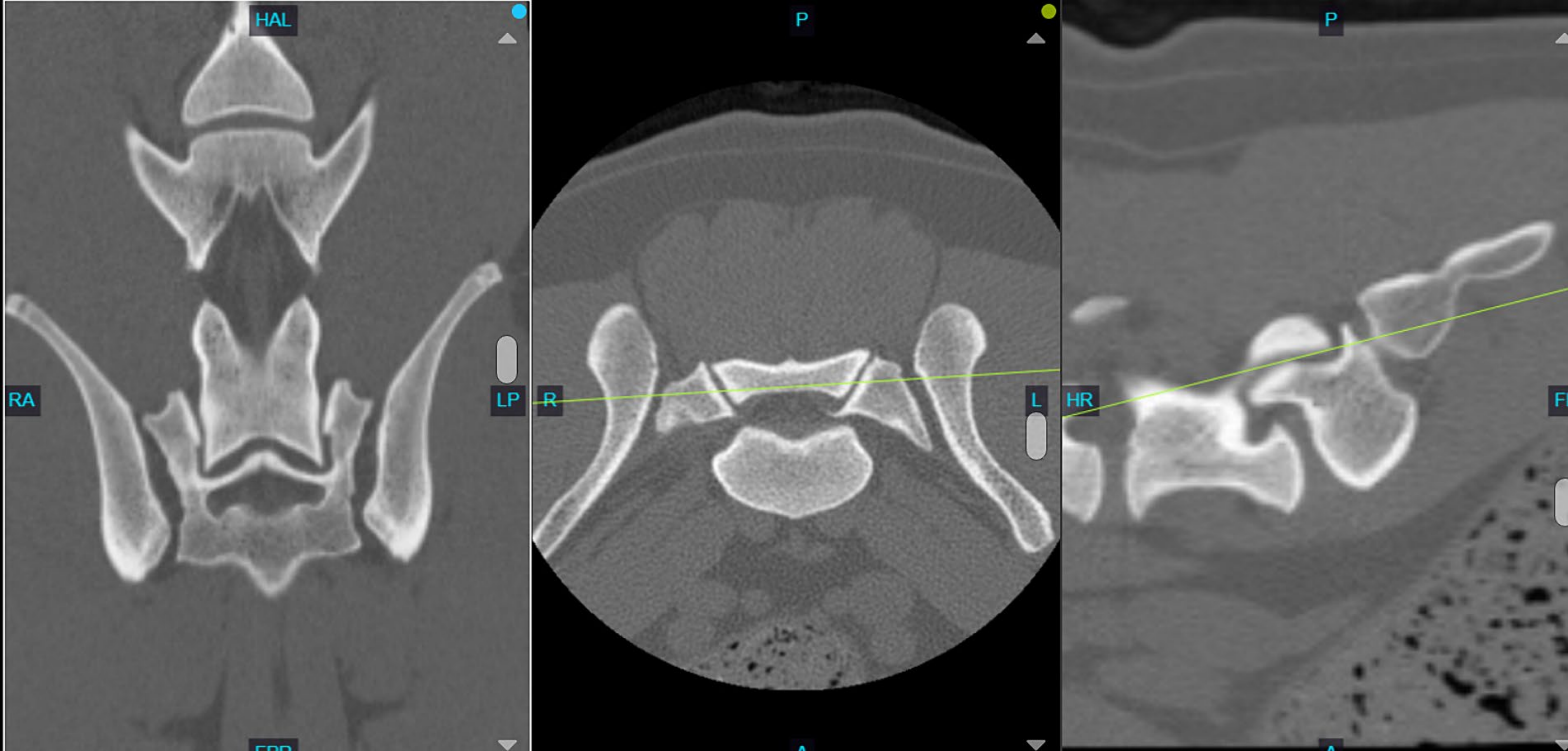
Computed tomography image of the L7-S1 joint showing the slice selection in the dorsal plane (left) with the concurrent transverse plane (middle) and sagittal plane (right). Note how the selected dorsal plane correlates to approximately the center of the LS articular process in the transverse plane and the relative contact point between the LS articular process from dorsal to ventral on the sagittal plane.

The imaging analysis protocol was developed by a surgery resident in consultation with an ACVS board-certified small animal surgeon (JTG). Using PACS software (Keystone Omni, Asteris, Montreal, Quebec, Canada), all measurements were acquired in an identical plane and orientation by selecting the DPCT slice that reflected the maximum LSAPD apparent in the frame, while the L7 and S1 articular processes could still be well visualized. The L7 articular process length was measured and defined as the distance (mm) from the caudal aspect of the L7 articular process to the cranial aspect of the L7 articular process (Figure 2A). The LSAPD is defined as the distance, in mm, from the cranial aspect of the L7 articular process to the cranial aspect of the sacral articular process (Figure 2B). The selected DPCT correlates with approximately the center of the LS articular process in the transverse plane and the relative contact point between the LS articular process from dorsal to ventral on the sagittal plane (Figure 3). To account for conformational differences beyond DLSS that could influence LSAPD, once LSAPD was quantified, an LSAPD: L7 ratio (LSDF ratio) was calculated by dividing the articular process displacement distance by the length of the L7 articular process as measured in the same plane.

### Data collection

A single observer (OC; surgery resident) evaluated all cases by performing each measurement on the left and right LS articular processes for each dog in triplicate. The slice location and orientation were not repeated for each triplicate measurement. To assess intra- and inter-observer reliability, images for each dog were masked to dog and group, with identification numbers ascribed for reassociation. A research randomizer program^1^ was used to identify 20 cases to be remeasured with the previously described method by the original observer (OC) for the assessment of intra-observer reliability. A second observer (JTG; ACVS board-certified small animal surgeon) performed measurements on 10 of the same randomly selected cases for the assessment of inter-observer reliability.

### Statistical analysis

All analyses were performed by a statistician (DK) using SAS 9.4 (Cary, NC), except for the calculation of ICCs, which were performed using the irr (Version 0.84.1, 2012) package in R (R Core Team). A significance threshold of 0.05 was used. The assumption of normality was evaluated via inspection of QQ- and PP-plots, histograms, and skewness. Normally distributed variables were summarized descriptively with mean and standard deviation (SD). Non-normally distributed variables were summarized descriptively with median and interquartile range (IQR). Age was compared between groups with a Mann–Whitney test and weight with a Welch’s *t*-test.

CT measurements were averaged by the dog prior to performing logistic regressions and calculating descriptive statistics, except for minimum and maximum, which were calculated on the unaveraged data. Linear mixed models (LMMs) and receiver operating characteristic (ROC) curves were performed on unaveraged data.

LMMs were used to compare articular process lengths between groups. The LMM had a single fixed factor of the group and a random intercept for each dog. QQ plots and histograms of conditional residuals confirmed the assumption of normality for LMMs. Satterthwaite degrees of freedom method and restricted maximum likelihood (REML) estimation method were used. Univariable and multivariable logistic regressions were used to test for an association between the odds of having DLSS with LSAPD and LSAPD ratio. Univariable models included a single covariate of LSAPD or LSAPD ratio and multivariable models additionally included covariates for age and weight. Log-likelihood *p*-values and odds ratios with profile-likelihood confidence limits were reported. ROC curves were constructed, and AUCs were calculated for flexed LSAPD and displacement ratios, which had *p* < 0.05. Hosmer and Lemeshow guidelines were used for the interpretation of AUC (0.7–0.8, acceptable; 0.8–0.9, excellent; and 0.9–1.0, outstanding diagnostic discrimination) (24).

A two-way random effects model with single observer/measurement and absolute agreement was used to calculate ICCs for inter- and intra-observer reliability. Koo guidelines based on 95% confidence intervals were used for the interpretation of ICC (<0.50 = poor, 0.50–0.75 = moderate, 0.75–0.90 = good, and > 0.90 = excellent) (25).

## Results

### Animals

A total of 54 dogs were enrolled and included in the data analysis: 30 dogs in the DLSS group and 24 dogs in the control group. Of the dogs in the DLSS group, 25 dogs were enrolled at BluePearl Pet Hospital, and 5 dogs were enrolled at Holland Military Working Dog Veterinary Hospital. Three dogs were originally included in the DLSS group after medical record review but excluded after imaging review, one diagnosed with a nerve root mass and two with severe LS articular process osteophytosis that precluded obtaining LSAPD measurements. A total of 28 DLSS dogs were presented for clinical signs suggestive of DLSS as noted by their owners or handlers, including painful or reluctant jumping, pelvic limb lameness after activity, or reduced activity level. Two MWDs included in the DLSS group had just been acquired, and clinical signs suggestive of DLSS were identified on clinical examination. Signs noted included pain on dorsal palpation of the lumbosacral vertebral column or tail extension. Demographic information for both groups is detailed in Table 1.

**Table 1:** Demographic data for included dogs with and without clinical symptoms of degenerative lumbosacral stenosis (DLSS).

	DLSS	Control	*p*-value
Total dogs, *n*	30	24	
Age (y), median (range)	4.5 (1.4–13)	1.4 (1.2–2.4)	<0.001*
Weight (kg), median (range)	33.1 (13.1–52.4)	33.5 (26.4–38.0)	0.554
Sex, *n*			
Male	8	19	
Castrated	10	-	
Female	4	-	
Spayed	8	4	
Breed, *n*			
American Staffordshire Terrier	1	-	
Australian Shepherd	1	-	
Belgian Malinois	1	12	
Border Collie	3	-	
Dutch Shepherd Dog	-	2	
German Shepherd Dog	9	10	
Golden Retriever	1	-	
Ibizan Hound	1	-	
Labrador Retriever	7	-	
Mixed Breed	6	-	

### Measurement reliability

Intra- and inter-observer reliabilities were good to excellent for all measurements (Table 2).

**Table 2:** Intra-and inter-observer reliability measures for dorsal plane computed tomography measurements obtained from 54 dogs with and without degenerative lumbosacral stenosis.

Measurements	Intra-observer ICC (95%CI)	Inter-observer ICC (95%CI)
Extension: L7 articular process, mm	0.93 (0.79–0.97)	0.92 (0.38–0.98)
Extension: LSAPD, mm	0.93 (0.79–0.98)	0.96 (0.91–0.99)
Extension: LSAPD ratio	0.88 (0.61–0.96)	0.95 (0.88–0.98)
Flexion: L7 articular process, mm	0.78 (0.45–0.93)	0.90 (0.59–0.97)
Flexion: LSAPD, mm	0.64 (0.28–0.84)	0.96 (0.80–0.99)
Flexion: LSAPD ratio	0.63 (0.26–0.83)	0.96 (0.78–0.99)

ICC, Intra-class correlation coefficient.

### Comparison between DLSS and control dogs

**Figure 4:**
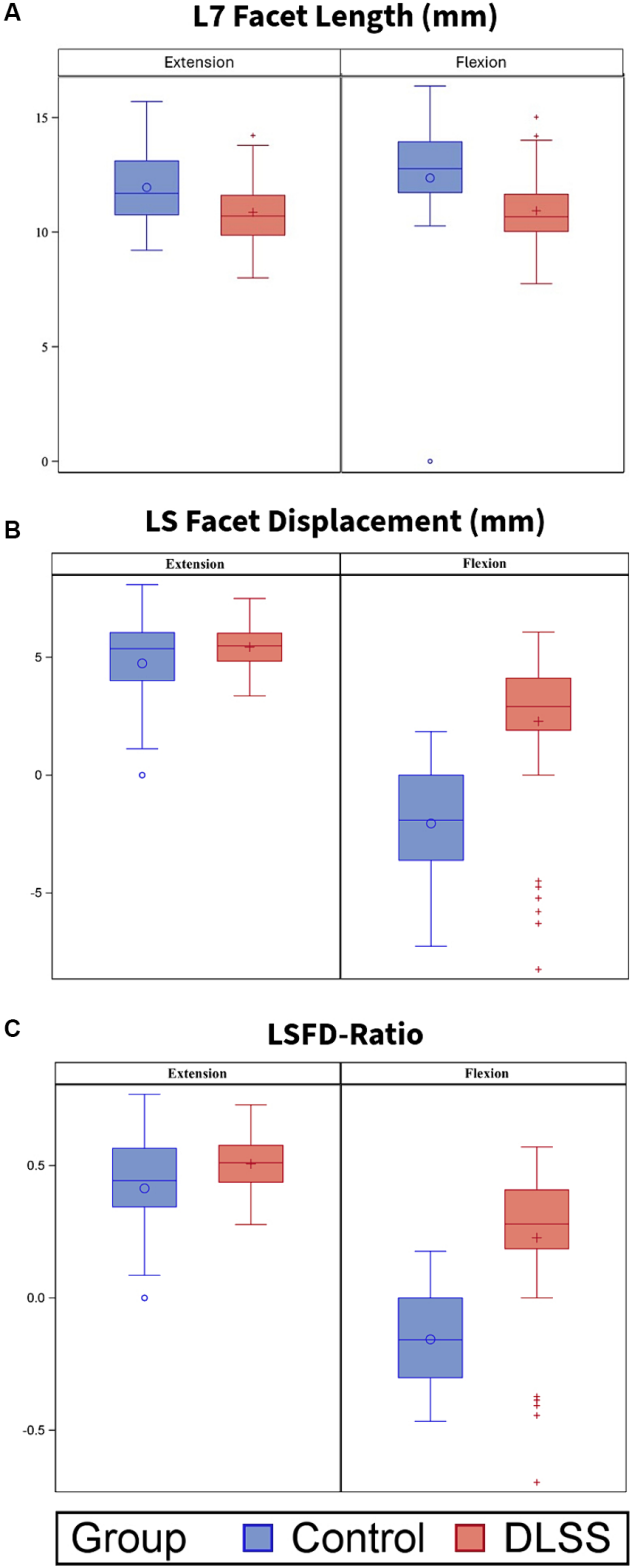
L7 articular process length (mm), Lumbosacral articular process displacement (LSAPD; mm) and LSAPD-ratio for dogs positioned in extended and flexed orientations for dorsal plane computed tomography (DPCT). Box and whisker plots depict median and interquartile range. **(A)** L7 articular process length was significantly greater in control dogs compared to those with DLSS in both extension (*p* = 0.004) and in flexion (*p* = 0.012). There were no significant differences between groups with respect to **(B)** LSAPD or **(C)** LSAPD-ratio in either position, although the DLSS group contained two visually distinct population with respect to measurement of LSAPD: a subset of dogs with LSAPD substantially higher than the median control dog, and a subset of dogs with LSAPD approximating or lower than LSAPD of the median control dog.

Measurements for the DLSS and control groups are reported in Table 3. The median flexed LSAPD in DLSS dogs was almost twice the median flexed LSAPD in control dogs (Figure 4).

**Table 3:** Descriptive statistics^a^ for dorsal plane computed tomography measurements obtained from 54 dogs with and without degenerative lumbosacral stenosis (DLSS) positioned in both flexion and extension.

Measurement	Group	*N*	Mean	Std dev	Median	Lower quartile	Upper quartile	Minimum	Maximum	*p*-value
Extension: L7 Articular process Length, mm	Control	24	12.0	1.4	12.0	10.7	13.1	9.2	15.7	0.004^b^
	DLSS	30	10.9	1.3	10.8	10.0	11.7	8.0	14.2	
Extension: LSAPD, mm	Control	24	4.7	2.1	5.2	4.2	6.1	0.0	8.1	0.146^c^
	DLSS	30	5.4	0.7	5.4	5.0	5.9	3.4	7.5	
Extension: LSAPD ratio	Control	24	0.4	0.2	0.4	0.4	0.5	0.0	0.8	0.051^c^
	DLSS	30	0.5	0.1	0.5	0.5	0.6	0.3	0.7	
Flexion: L7 Articular process Length, mm	Control	24	12.3	1.9	12.5	11.2	13.8	0.0	16.4	0.009^b^
	DLSS	30	11.1	1.5	10.8	10.2	11.8	7.8	15.0	
Flexion: LSAPD, mm*	Control	24	−2.1	2.2	−1.5	−3.7	0.0	−7.3	1.8	<0.001^c^
	DLSS	30	2.3	3.1	2.9	2.2	4.1	−8.2	6.1	
Flexion, LSAPD ratio*	Control	24	−0.2	0.2	−0.1	−0.3	0.0	−0.5	0.2	<0.001^c^
	DLSS	30	0.2	0.3	0.3	0.2	0.4	−0.7	0.6	

Bilateral measurements were averaged per dog prior to descriptive statistics except for minimum and maximum, which were calculated on the un-averaged values.

^a^Both sides per dog averaged prior to descriptive statistics except for minimum and maximum which were calculated on the un-averaged values.

^b^Linear mixed model.

^c^Log-likelihood ratio test.

^*^Non-normally distributed.

LSAPD, lumbosacral articular process displacement.

### Diagnostic discrimination

**Figure 5:**
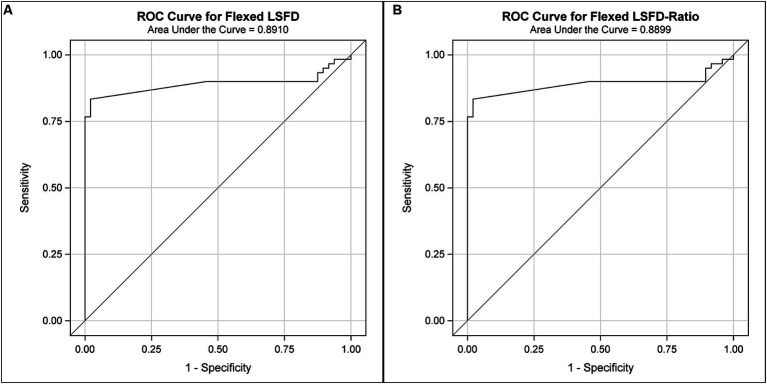
Receiver operator curves (ROCs) for the diagnostic sensitivity and specificity of DPCT measurements in the diagnosis of DLSS. For LSAPD as measured in a flexed position **(A)**, the area under the ROC curve (AUC) (95%CI) was 0.89 (0.82–0.96), which indicates excellent to outstanding diagnostic discrimination for DLSS. For the LSAPD ratio as measured in a flexed position **(B)**, the area under the ROC curve (AUC) (95%CI) was 0.89 (0.82–0.96), which indicates excellent to outstanding diagnostic discrimination for DLSS.

The area under the curve (AUC) (95%CI) for LSAPD and LSAPD ratio measured in a flexed position were both 0.89 (0.82–0.96), suggesting potentially excellent discrimination for utilizing this measurement as a marker for diagnosing DLSS in the current cohort (Figure 5). The cutoff for LSAPD measured in a flexed position that optimally balances the tradeoff between sensitivity (83%) and specificity (98%) for diagnosis was ≥1.2 mm (Table 4). The cutoff for the LSAPD ratio measured in a flexed position that optimally balances the tradeoff between sensitivity (83%) and specificity (98%) was ≥0.09 (Table 5). In comparison, diagnostic discrimination for DLSS using the LSAPD ratio for dogs in an extended position was unacceptable, where the AUC (95%CI) was 0.63 (0.52–0.74) (24).

**Table 4:** Diagnostic sensitivity and specificity for LSAPD, as measured in a flexed position, in a population of 54 dogs with and without DLSS for selected cutoffs.

Flexion LSAPD cutoff	Sensitivity	Specificity
≥6.1	2%	100%
≥3.0	50%	100%
≥2.0	73%	100%
≥1.2*	83%	98%
0.0	90%	54%
−1.8	90%	52%
−5.6	95%	8%
−8.2	100%	0%

When giving equal weight to sensitivity and specificity, the optimal cutoff is ≥1.2 mm. Other cutoffs prioritize sensitivity or specificity, either of which may be preferable in clinical use. *Highest Youden’s Index.

**Table 5:** Diagnostic sensitivity and specificity for LSAPD ratio, as measured in a flexed position, in a population of 54 dogs with and without DLSS for selected cutoffs.

Flexion LSAPD ratio cutoff	Sensitivity	Specificity
≥18%	77%	100%
≥9%*	83%	98%
≥0%	90%	54%
≥ − 37%	92%	10%
≥ − 39%	95%	10%
≥ − 41%	97%	8%
≥ − 44%	98%	4%
≥ − 70%	100%	0%

When giving equal weight to sensitivity and specificity, the optimal cutoff is ≥9%. Other cutoffs prioritize sensitivity or specificity, either of which may be preferable in clinical use. *Highest Youden’s Index.

In the univariable analysis, increasing LSAPD was significantly associated with a diagnosis of DLSS, where the odds of a clinical diagnosis of DLSS increased by 80% per 1 mm increase in LSAPD in LS flexion (OR (95%CI) 1.8 (1.3–2.3), *p* < 0.0001). Furthermore, the odds of a clinical diagnosis of DLSS were two times higher for every 0.1 increase in LSAPD ratio in LS flexion upon univariable analysis (OR (95%CI) 2.0 (1.5–2.8), *p* < 0.0001). A similar statistically significant relationship was not identified via univariable analysis for LSAPD (OR (95%CI) 1.4 (0.9–2.3), *p* = 0.146) or LSAPD ratio (OR (95%CI) 1.6 (1.0–2.7), *p* = 0.051) in LS extension with the diagnosis of DLSS. A relatively small number of cases per group supported the inclusion of only two covariates in multivariable analysis to avoid overfitting. When age and weight were subsequently included as covariates in a multivariable analysis, a significant relationship between LSAPD or LSAPD ratio and odds of diagnosis of DLSS was not demonstrated for either position, although all odds ratio estimates were > 1 (Table 6).

**Table 6:** Odds ratios for the association of dorsal plane computed tomography measurements with the odds of degenerative lumbosacral stenosis from univariable and multivariable logistic regressions.

Measurement	Univariable		Multivariable	
	OR (95% CI)	*p*-value	OR (95% CI)	*p*-value
Extension: LSAPD, per mm	1.4 (0.9–2.3)	0.146	1.3 (0.7–3.6)	0.537
Flexion: LSAPD, per mm	1.8 (1.3–2.3)	<0.0001	1.4 (0.9–2.6)	0.109
Extension, LSAPD ratio, per 10%	1.6 (1.0–2.7)	0.051	1.5 (0.7–4.7)	0.316
Flexion, LSAPD ratio, per 10%	2.0 (1.5–2.8)	<0.0001	1.5 (0.7–3.2)	0.119

OR, odds ratio.

## Discussion

Our results suggest that the evaluation of CT images of the lumbosacral vertebral column in a dorsal plane provides an easy approach for measuring LSAPD that yields consistent values when obtained by different reviewers and might hold promise as an imaging marker of DLSS in a clinical setting. Specifically, diagnostic discrimination was maximized in the present cohort when a cutoff value of >1.2 mm for LSAPD or > 9% for LSAPD ratio was applied to measurements acquired with dogs positioned in a flexed orientation, providing an important candidate parameter to carry forward in future prospective studies and evaluating the positive predictive value of this measurement in dogs with a clinical suspicion of DLSS; however, based on multivariable analysis, we were unable to demonstrate a difference between DLSS-affected and control dogs with respect to LSAPD, suggesting that a larger prospective controlled study is needed.

In the present study, 52 out of 54 dogs had positive LSAPD in LS extension regardless of clinical DLSS status; however, all control dogs had LSAPD values that were negative or zero in LS flexion, except for one that had a small positive LSAPD. A negative articular process displacement value indicates the displacement of the cranial aspect of the sacral articular process in a caudal direction when compared to the cranial aspect of the L7 articular process. Moreover, a greater difference in LSAPD between flexed and extended positioning in the DLSS group could correlate with instability or variability in the LS joint of the latter group. It is not known if the displacement of the LS articular processes found in this study is normal in the extended canine vertebral column, though Lampe et al. did not report LS misalignment or articular process displacement on dynamic MRI examinations of dogs with clinically normal LS spines (26). Chronic LSAPD may promote LS articular process joint capsule fibrosis and thickening through repetitive stretching of the joint capsule, preventing the reduction of the LS articular process to either 0 or negative LSAPD, such that small displacement in the flexed position can translate to a more relevant finding. Based on the results from this study, the formulating thought is that in extension, LSAPD will be present to some degree in most unaffected DLSS dogs, and in flexion, LSAPD reduction should return to complete alignment in most unaffected DLSS dogs.

There were several limitations associated with this study. Multivariable analyses suggest there were confounding factors impacting the significance and magnitude of initially identified differences between groups. Given the challenges with obtaining advanced imaging of the vertebral column in healthy pet dogs, a population of healthy military working dogs undergoing screening CTs was utilized as a control population and therefore was imaged at a different facility from DLSS cases. Moreover, some dogs with subclinical DLSS might have been included in the control group, given the incidence of subclinical disease in these dogs (21). There were also differences in age between the two groups, where DLSS dogs were significantly older than control dogs. Finally, protocols for chemical restraint for imaging differed between the two groups, although dogs in both groups were placed in a plane of heavy sedation for imaging. We cannot exclude that some differences in imaging measurements between groups were influenced or exacerbated by factors related to imaging location and setup, positioning, or age-related differences in vertebral column anatomy; however, patient positioning was standardized across facilities, and clinical signs of DLSS were inherently more common in middle-aged to older large breed dogs, making age matching a challenge. Our results suggest that the diagnostic discrimination of LSAPD and LSAPD ratio, specifically as measured with the lumbosacral junction in a flexed position, warrants further investigation as an imaging marker of DLSS in a larger, prospective study, where sedation protocol, inclusion criteria for control and affected groups, and imaging setup (performed in one facility) are more stringently controlled.

In conclusion, the quantification of LSAPD in flexion and extension on DPCT is reliable within and between evaluators, and LSAPD warrants further explanation as an imaging diagnostic tool for identifying dogs with early signs of DLSS with instability. If ultimately validated as an imaging biomarker, this measure could be useful as a screening test for working dogs and others at high risk for DLSS and could be useful for guiding decision-making for treatment intervention. Future prospective studies are indicated to further explore this diagnostic imaging marker in clinical cases suspected of having DLSS and non-clinical cases for comparison.

## Data availability statement

The raw data supporting the conclusions of this article will be made available by the authors, without undue reservation.

## Ethics statement

The animal studies were approved by the BluePearl Science Veterinary Clinical Studies Committee and the Holland Military Working Dog Veterinary Hospital Institutional Animal Care Use Committee. The studies were conducted in accordance with the local legislation and institutional requirements. Written informed consent was obtained from the owners for the participation of their animals in this study.

## Author contributions

OC: Data curation, Investigation, Methodology, Validation, Writing – original draft, Writing – review & editing. SF: Data curation, Methodology, Project administration, Supervision, Writing – original draft, Writing – review & editing. DK: Data curation, Formal analysis, Writing – original draft, Writing – review & editing. SM: Data curation, Supervision, Writing – original draft, Writing – review & editing. JG: Conceptualization, Investigation, Methodology, Supervision, Writing – original draft, Writing – review & editing.

## Footnotes

^1^http://randomizer.org
